# Novel Carbapenemases FLC-1 and IMI-2 Encoded by an Enterobacter cloacae Complex Isolated from Food Products

**DOI:** 10.1128/AAC.02338-18

**Published:** 2019-05-23

**Authors:** Michael S. M. Brouwer, Kamaleddin H. M. E. Tehrani, Michel Rapallini, Yvon Geurts, Arie Kant, Frank Harders, Vida Mashayekhi, Nathaniel I. Martin, Alex Bossers, Dik J. Mevius, Ben Wit, Kees T. Veldman

**Affiliations:** a Department of Bacteriology and Epidemiology, Wageningen Bioveterinary Research, Lelystad, The Netherlands; b Biological Chemistry Group, Institute of Biology Leiden, Leiden University, Leiden, The Netherlands; c Netherlands Food and Consumer Product Safety Authority (NVWA), Wageningen, The Netherlands; d Department of Infection Biology, Wageningen Bioveterinary Research, Lelystad, The Netherlands; e Department of Infectious Diseases and Immunology, Utrecht University, Utrecht, The Netherlands; f Molecular Targeted Therapies Group, Cell Biology Division, Department of Biology, Utrecht University, Utrecht, The Netherlands

**Keywords:** antimicrobial resistance, carbapenemase, *Enterobacter*, plasmid

## Abstract

Food for human consumption is screened widely for the presence of antibiotic-resistant bacteria to assess the potential for transfer of resistant bacteria to the general population. Here, we describe an Enterobacter cloacae complex isolated from imported seafood that encodes two carbapenemases on two distinct plasmids. Both enzymes belong to Ambler class A β-lactamases, the previously described IMI-2 and a novel family designated FLC-1. The hydrolytic activity of the novel enzyme against aminopenicillins, cephalosporins, and carbapenems was determined.

## Abstract (precis)

Food for human consumption is screened widely for the presence of antibiotic-resistant bacteria to assess the potential for transfer of resistant bacteria to the general population. Here, we describe an Enterobacter cloacae complex isolated from imported seafood that encodes two carbapenemases on two distinct plasmids.

## INTRODUCTION

To combat antimicrobial resistance (AMR) effectively, it is important to monitor reservoirs that may be sources of transmission to humans. Relevant reservoirs are those that may be attributed to the AMR genes found in the general population and patients. Seafood has been implicated as a potential source of AMR genes entering populations when several aquatic bacteria carrying carbapenemase genes were identified in seafood imported from Southeast Asia (1, 2). Often, these genes are chromosomally located in nonpathogenic aquatic bacterial species, limiting them as relevant threats for the general population (3). However, more recent studies screening seafood imported from Southeast Asia have found carbapenemases encoded in human pathogens or on conjugative plasmids (4–6). As such, seafood imported from countries with high carbapenemase prevalence may need to be included in monitoring programs.

Proteins with carbapenemase activity fall into the three major Ambler classes A, B, and D β-lactamases (7). Genes of these classes have been described on mobile genetic elements, such as plasmids and chromosomally integrated elements, which adds to the concerns regarding these genes because they facilitate the spread of these genes among both commensal and pathogenic bacteria (6, 8, 9). The family of Enterobacteriaceae consists of many commensal, opportunistic, and infectious species that can readily exchange genetic material. The organisms are collectively referred to as carbapenemase-producing *Enterobacteriaceae* when they have acquired and express one of these genes.

Recently, Enterobacter cloacae complex and Vibrio cholerae isolates have been described with a distinctive phenotype of hydrolyzing penicillins, aztreonam, and carbapenems but not extended-spectrum cephalosporins (10).

In March 2017, we isolated an E. cloacae complex isolate, designated 3442, on a ChromID Carba plate (bioMérieux Benelux BV) from a sample of frozen vannamei white shrimp (Litopenaeus vannamei) originating in India. Species identification was performed using matrix-assisted laser desorption ionization–time of flight mass spectrometry (Bruker Microflex LT/SH, Bruker Daltonics, Billerica MA). Susceptibility testing was performed with broth microdilution in the Sensititre panels EUVSEC and EUVSEC2 (Thermo Fisher, Waltham MA) and interpreted using epidemiological cutoff values for cephalosporins and carbapenems as defined for Escherichia coli by EUCAST. The isolate exhibited an unusual phenotype, i.e., non-wild type susceptible to carbapenems (meropenem, ertapenem, and imipenem) and susceptible to extended-spectrum cephalosporins (cefotaxime, ceftazidime, and cefepime) (Table 1).

**TABLE 1:** MICs of E. cloacae complex 3442, E. coli recipients, transformant, and transconjugant of pIMI2 and transformant of pBAD-FLC

Antibiotic	MIC (μg/ml) for:						
	E. cloacae 3442	E. coli DH10B pIMI2 (transformant)	E. coli DH10B	E. coli E3110 pIMI2 (transconjugant)	E. coli E3110	E. coli LMG194 pBAD-FLC	E. coli LMG194
Ampicillin	>64	>64	4	>64	4	>64	4
Cefotaxime	≤0.25	≤0.25	≤0.25	≤0.25	≤0.25	2	≤0.25
Cefotaxime/clavulanic acid	0.25/4	≤0.06/4	≤0.06/4	0.12/4	0.12/4	0.12/4	≤0.06/4
Ceftazidime	≤0.5	0.5	0.5	0.5	0.5	1	0.5
Ceftazidime/clavulanic acid	≤0.12/4	0.25/4	0.25/4	0.25/4	0.25/4	0.25/4	0.25/4
Cefepime	0.12	0.25	≤0.06	0.25	0.12	0.5	0.12
Cefoxitin	64	16	8	8	16	8	8
Ertapenem	>2	>2	≤0.015	>2	≤0.015	>2	≤0.015
Imipenem	>16	16	0.25	>16	0.5	16	0.25
Meropenem	>16	8	≤0.03	>16	0.06	4	≤0.03
Temocillin	4	16	32	16	16	16	8

Complete genomic DNA was isolated using the Gentra Puregene kit (Qiagen), and whole-genome sequencing was performed using Illumina MiSeq PE300 and ONT MinIon sequencing. Hybrid assemblies were created using a SPAdes reconstructed genome consisting of one chromosome and three plasmids. The chromosome data were used to determine the MLST type as ST813, a sequence type first described last year in companion animals in Japan (11).

Plasmid p3442-FLC-1 is 93 kb and carries a novel carbapenemase with close sequence similarity to *bla*_FRI-1_ (10). Plasmid p3442-IMI2 is 78 kb and carries the carbapenemase *bla*_IMI-2_. Both plasmids are closely related to the IncFII plasmids first described in *Yersinia* spp. but have new FII-Y alleles (submitted to pubMLST and designated FII-Y-9 and FII-Y-10) (12). The closest relative to both plasmids is pIMI-6, described in an E. cloacae complex clinical isolate from Canada, which carries the carbapenemase *bla*_IMI-6_ (8). The two IncFII-Y plasmids contain much overlap in their sequences, but both plasmids appear to have lost significant amounts of genetic material, and we hypothesize that these plasmids developed from a pair of complete IncFII-Y plasmids. Currently, the two plasmids together contain most of the functions of IncFII-Y plasmids, as shown in Fig. S1 in the supplemental material in a BLAST ring image generator (13) comparison with the E. cloacae complex plasmid pIMI-6 (8), although several regions are absent in both plasmids.

To assess the mobility of these plasmids, transformation and conjugation experiments were performed as previously described (14). Carbapenem-resistant transconjugants and transformants were tested for *bla*_IMI_ and *bla*_FRI_. Out of >200 colonies, only *bla*_IMI_-positive transconjugants and transformants were detected. Antibiograms of transformants and transconjugants are included in Table 1. Whereas only p3442-IMI2 may be both transformed and conjugated into E. coli recipient cells, p3442-FLC-1 may be transferred at lower frequencies, below the level of detection used in the current experiments.

Although *bla*_IMI-2_ transformants and transconjugants were carbapenem resistant, we hypothesized that the *bla*_FRI_-related gene may also have carbapenemase activity. Because the plasmid carrying the gene could not be transformed or conjugated into E. coli cells, the gene was cloned into an arabinose-inducible expression vector, pBAD-FLC (Vectorbuilder), and expressed in E. coli LMG194 (pBAD TOP TA expression kit, Invitrogen, Saint-Aubin, France) (15). The MIC of E. coli LMG194 pBAD-FLC was determined by broth microdilution after culture overnight in RPMI medium plus 0.2% glucose followed by dilution in Mueller-Hinton broth containing 0.2% arabinose and incubation at 37°C for 1 h to enable expression to start. Standard protocols were followed thereafter and E. coli LMG194 and ATCC 25922 were used as negative controls. E. coli LMG194 pBAD-FLC showed resistance against carbapenems and extended-spectrum cephalosporins (Table 1). The FRI variant was concluded to be a carbapenemase and is further referred to as FRI-like carbapenemase-1 (*bla*_FLC-1_).

**FIG 1:**
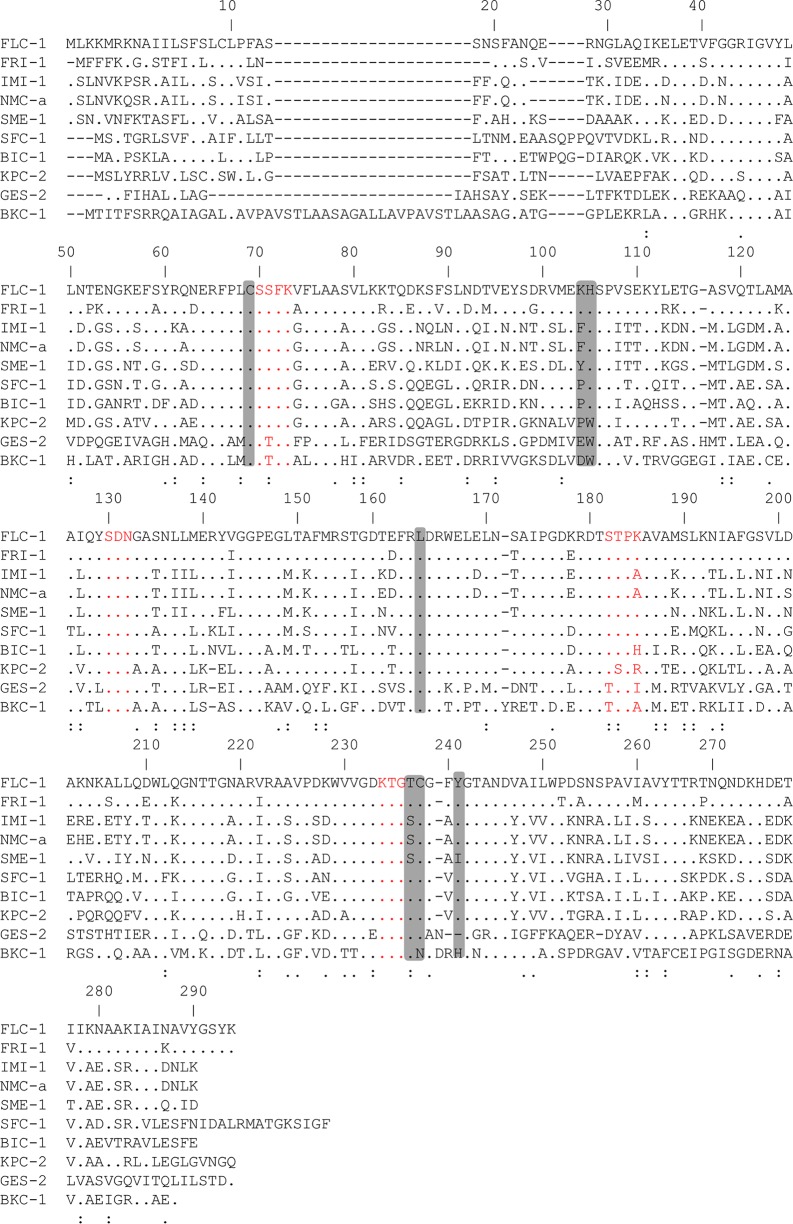
Multiple sequence alignment of the amino acid sequences of class A carbapenemases. Residues that are identical to the sequence of FLC-1 are shown as a period in the sequence; a dash indicates a gap that was inserted during alignment. A colon underneath the sequence indicates a substitution of strong similar properties, and a dot under the sequence indicates a substitution of a weak similar property. Numbering of the amino acids was done according to the method described for class A β-lactamases by Ambler et al. (24). Residues conserved among class A β-lactamases are shown in red, and residues conserved among class A carbapenemases are shaded in gray.

**FIG 2:**
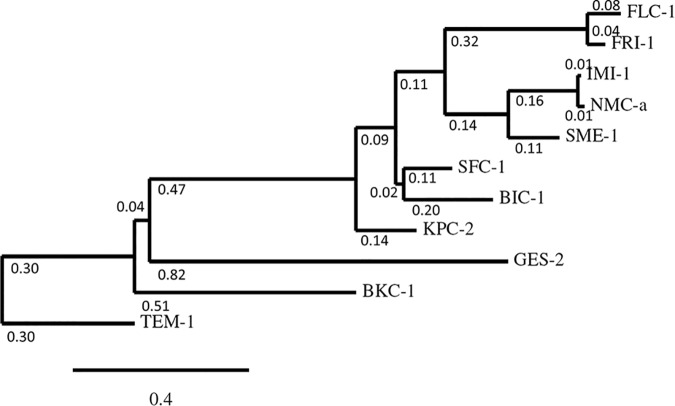
Phylogram of FLC-1 and 10 representative class A β-lactamases. Amino acid sequences were analyzed by Clustal Omega using the neighbor-joining method. Branch lengths are proportional to the number of amino acid changes.

Multiple sequence alignments were made comparing FLC-1 with several members of plasmid-encoded Ambler class A carbapenemases (Fig. 1). All conserved residues among class A β-lactamases were present. The most related protein family was that of the French imipenemase (FRI), with 82% identity to FRI-1 and 87% to FRI-5 (10, 16, 17) (Fig. 2).

As previously described for other Ambler class A carbapenemases, *bla*_FLC-1_ is preceded by a lysR-type regulator, transcribed in the opposite direction, which is predicted to regulate the expression of the protein (18). This region was flanked by the remnants of two insertion elements, related to IS*3* and IS*Ec25*, which were likely responsible for the integration of the region onto an ancestor of the plasmid, which is also common for Ambler class A carbapenemases (8).

The soluble protein fractions of arabinose-induced E. coli LMG194 pBAD-FLC and E. coli LMG194 were prepared as described in the supplemental material, and their biochemical properties were evaluated. Analysis of the periplasmic protein fractions by SDS-PAGE showed induction of a protein between 25 and 35 kDa as expected (FLC-1 molecular weight, ∼33 kDa; see Fig. S2 in the supplemental material). Hydrolysis of various β-lactam antibiotics was monitored with a Spark microplate reader (Tecan) at 23°C using 96-well UV-Star microplates. Phosphate-buffered saline (0.01% Triton X-100, pH 7.4) was used as the assay buffer. The extinction coefficients for the β-lactam antibiotics studied were Δε_235_ = 900 M^−1^ cm^−1^ for ampicillin, Δε_297_ = 10,940 M^−1^ cm^−1^ for meropenem, Δε_295_ = 11,500 M^−1^ cm^−1^ for imipenem, Δε_300_ = 6,920 M^−1^ cm^−1^ for ertapenem, and Δε_264_ = 7,250 M^−1^ cm^−1^ for cefotaxime. To calculate kinetic parameters, including *K_m_* and *V*_max_, the measured initial velocities of the hydrolysis of the substrates were fit into the Michaelis-Menten equation using GraphPad Prism 7 software (see Fig. S3 in the supplemental material for Michaelis-Menten curves). Initially, cytoplasmic fractions of E. coli containing the plasmid showed hydrolysis of nitrocefin, while cytoplasmic fractions of E. coli lacking the plasmid did not (see Fig. S4a in the supplemental material). Expanding these measurements to several β-lactam antibiotics over time allowed for the determination of kinetic parameters of the protein-expressing cells (Table 2). The enzymatic activity of FLC-1 clearly showed greater efficiency of the enzyme toward carbapenems than toward cephalosporins (as evident by the relative *k*_cat_/*K_m_* values) (Table 2), with activity against ceftazidime and cefepime below the threshold of detection. Using nitrocefin as the substrate, the inhibition of FLC-1 enzymatic activity by clavulanic acid was tested, and the 50% inhibitory concentration was calculated (1.974 ± 0.090 μM) (Fig. S4b).

**TABLE 2:** Kinetic parameters determined for the cytoplasmic fraction of E. coli LMG-194 producing FLC-1

Antibiotic	Protein concn (μg · ml)^*a*^^,^^*b*^	*K_m_* (μM)	*V*_max_/μg protein^*c*^	Relative *k*_cat_/*K_m_*
Ampicillin	5.53	1,649 ± 174.2	(1,490 ± 70) × 10^−3^	1.00
Meropenem	100	32.4 ± 9.3	(2.05 ± 0.14) × 10^−3^	0.07
Imipenem	17.68	177.2 ± 12.5	(48.61 ± 1.40) × 10^−3^	0.30
Ertapenem	44.21	29.6 ± 11.7	(6.34 ± 0.67) × 10^−3^	0.24
Cefotaxime	106.1	377.1 ± 110.6	(7.85 ± 1.25) × 10^−3^	0.02
Ceftazidime		ND^*d*^	<65 × 10^−3^^*e*^	
Cefepime		ND^*d*^	<34 × 10^−3^^*e*^	

a Protein concentration of the cytoplasmic fraction.

b The E. coli strain producing FLC and the nontransformed strain were used to prepare cytoplasmic fractions. The highest tested concentration of both preparations was 176.83 μg/ml. None of the tested antibiotics were hydrolyzed by the nontransformed E. coli cytoplasmic fraction.

c Expressed as μM/s/μg of protein.

d Not determinable.

e Because no substrate hydrolysis was detected, the *V*_max_ data for ceftazidime and cefepime have been reported as less than the limit of detection/μg protein.

Class A carbapenemases include members of GES, KPC, SME, and IMI/NMC-A enzymes plus SFC-1 and SHV-38 (19). With the exception of GES-1, most class A carbapenemases demonstrate higher carbapenemase activity of various degrees relative to extended-spectrum β-lactamases (19–22). FRI-1 is the closest member of the class A carbapenemases relative to FLC-1 and was found to be at least 15 times more efficient in degrading carbapenems than extended-spectrum cephalosporins (10). Here, we report a similar substrate preference for the FLC-1 enzyme, which hydrolyzes imipenem, ertapenem, and meropenem with greater efficiency than the cephalosporins tested (Table 2).

To control AMR and retain effective use of antimicrobials in human and veterinary medicine, a complete and correct overview of the impact that these human and animal reservoirs have on each other is essential. *bla*_FLC-1_ was detected here in a sample of raw shrimp from India, but members of the FRI family, to which FLC is most closely related, and IMI, NMC-a, and SME have been described in a various global reservoirs (8, 10, 16, 17, 21, 23). Reliable databases of acquired resistance genes and point mutations leading to resistance are essential to determine the gene responsible for a particular resistant phenotype. The complete analysis presented here of the novel carbapenemase FLC-1 in its complete genetic carrier context will aid in the future for the recognition of its gene, *bla*_FLC-1_, and related carbapenemases.

### Accession numbers.

The whole-genome sequence of isolate 3442 was submitted to GenBank, and the chromosome and individual plasmids are available under accession numbers CP033466 to CP033469. The protein sequence of *bla*_FLC-1_ was submitted to GenBank under accession number ATX60370.1.

## Supplementary Material

Supplemental file 1

## References

[1] Rubin JE, Ekanayake S, Fernando C 2014 Carbapenemase-producing organism in food, 2014. Emerg Infect Dis 20:1264–1265. doi:10.3201/eid2007.140534. 24960459 PMC4073846

[2] Morrison BJ, Rubin JE 2015 Carbapenemase producing bacteria in the food supply escaping detection. PLoS One 10:e0126717. doi:10.1371/journal.pone.0126717. 25966303 PMC4429064

[3] Ceccarelli D, van Essen-Zandbergen A, Veldman KT, Tafro N, Haenen O, Mevius DJ 2017 Chromosome-based *bla*_OXA-48_-like variants in *Shewanella* species isolates from food-producing animals, fish, and the aquatic environment. Antimicrob Agents Chemother 61:e01013-16. doi:10.1128/AAC.01013-16. 27855066 PMC5278689

[4] Mangat CS, Boyd D, Janecko N, Martz SL, Desruisseau A, Carpenter M, Reid-Smith RJ, Mulvey MR 2016 Characterization of VCC-1, a novel Ambler class A carbapenemase from *Vibrio cholerae* isolated from imported retail shrimp sold in Canada. Antimicrob Agents Chemother 60:1819–1825. doi:10.1128/AAC.02812-15. 26824956 PMC4775928

[5] Roschanski N, Guenther S, Vu TTT, Fischer J, Semmler T, Huehn S, Alter T, Roesler U 2017 VIM-1 carbapenemase-producing *Escherichia coli* isolated from retail seafood, Germany 2016. Euro Surveill 22. doi:10.2807/1560-7917.ES.2017.22.43.17-00032. PMC5718389 29090680

[6] Brouwer MSM, Rapallini M, Geurts Y, Harders F, Bossers A, Mevius DJ, Wit B, Veldman KT 2018 *Enterobacter cloacae* complex isolated from shrimps from Vietnam carrying *bla*_IMI-1_ resistant to carbapenems but not cephalosporins. Antimicrob Agents Chemother 62:e00398-18. doi:10.1128/AAC.00398-18. 29686153 PMC6021663

[7] Nordmann P, Naas T, Poirel L 2011 Global spread of carbapenemase-producing Enterobacteriaceae. Emerg Infect Dis 17:1791–1798. doi:10.3201/eid1710.110655. 22000347 PMC3310682

[8] Boyd DA, Mataseje LF, Davidson R, Delport JA, Fuller J, Hoang L, Lefebvre B, Levett PN, Roscoe DL, Willey BM, Mulvey MR 2017 *Enterobacter cloacae* complex isolates harboring *bla*_NMC-A_ or *bla*_IMI_-type class A carbapenemase genes on novel chromosomal integrative elements and plasmids. Antimicrob Agents Chemother 61:e02578-16. doi:10.1128/AAC.02578-16. 28223374 PMC5404547

[9] Walsh TR, Toleman MA, Poirel L, Nordmann P 2005 Metallo-beta-lactamases: the quiet before the storm? Clin Microbiol Rev 18:306–325. doi:10.1128/CMR.18.2.306-325.2005. 15831827 PMC1082798

[10] Dortet L, Poirel L, Abbas S, Oueslati S, Nordmann P 2015 Genetic and biochemical characterization of FRI-1, a carbapenem-hydrolyzing class A beta-lactamase from *Enterobacter cloacae*. Antimicrob Agents Chemother 59:7420–7425. doi:10.1128/AAC.01636-15. 26392482 PMC4649213

[11] Harada K, Shimizu T, Mukai Y, Kuwajima K, Sato T, Kajino A, Usui M, Tamura Y, Kimura Y, Miyamoto T, Tsuyuki Y, Ohki A, Kataoka Y 2017 Phenotypic and molecular characterization of antimicrobial resistance in Enterobacter spp. isolates from companion animals in Japan. PLoS One 12:e0174178. doi:10.1371/journal.pone.0174178. 28328967 PMC5362103

[12] Villa L, Garcia-Fernandez A, Fortini D, Carattoli A 2010 Replicon sequence typing of IncF plasmids carrying virulence and resistance determinants. J Antimicrob Chemother 65:2518–2529. doi:10.1093/jac/dkq347. 20935300

[13] Alikhan NF, Petty NK, Ben Zakour NL, Beatson SA 2011 BLAST ring image generator (BRIG): simple prokaryote genome comparisons. BMC Genomics 12:402. doi:10.1186/1471-2164-12-402. 21824423 PMC3163573

[14] Liakopoulos A, Geurts Y, Dierikx CM, Brouwer MS, Kant A, Wit B, Heymans R, van Pelt W, Mevius DJ 2016 Extended-spectrum cephalosporin-resistant Salmonella enterica serovar Heidelberg strains, the Netherlands(1). Emerg Infect Dis 22:1257–1261. doi:10.3201/eid2207.151377. 27314180 PMC4918182

[15] Guzman LM, Belin D, Carson MJ, Beckwith J 1995 Tight regulation, modulation, and high-level expression by vectors containing the arabinose PBAD promoter. J Bacteriol 177:4121–4130. doi:10.1128/jb.177.14.4121-4130.1995. 7608087 PMC177145

[16] Meunier D, Findlay J, Doumith M, Godoy D, Perry C, Pike R, Gronthoud F, Shryane T, Poirel L, Welfare W, Woodford N, Hopkins KL 2017 FRI-2 carbapenemase-producing Enterobacter cloacae complex in the UK. J Antimicrob Chemother 72:2478–2482. doi:10.1093/jac/dkx173. 28605515

[17] Kubota H, Uwamino Y, Matsui M, Sekizuka T, Suzuki Y, Okuno R, Uchitani Y, Ariyoshi T, Aoki W, Suzuki S, Kuroda M, Shinkai T, Yokoyama K, Sadamasu K, Funakoshi T, Murata M, Hasegawa N, Iwata S 2018 FRI-4 carbapenemase-producing Enterobacter cloacae complex isolated in Tokyo, Japan. J Antimicrob Chemother 72:2478–2482. doi:10.1093/jac/dkx173. 30060114

[18] Naas T, Nordmann P 1994 Analysis of a carbapenem-hydrolyzing class-a beta-lactamase from Enterobacter-Cloacae and of its Lysr-type regulatory protein. Proc Natl Acad Sci U S A 91:7693–7697. doi:10.1073/pnas.91.16.7693. 8052644 PMC44468

[19] Walther-Rasmussen J, Høiby N 2007 Class A carbapenemases. J Antimicrob Chemother 60:470–482. doi:10.1093/jac/dkm226. 17595289

[20] Queenan AM, Bush K 2007 Carbapenemases: the versatile beta-lactamases. Clin Microbiol Rev 20:440–458, table of contents. doi:10.1128/CMR.00001-07. 17630334 PMC1932750

[21] Rasmussen BA, Bush K, Keeney D, Yang Y, Hare R, O'Gara C, Medeiros AA 1996 Characterization of IMI-1 beta-lactamase, a class A carbapenem-hydrolyzing enzyme from *Enterobacter cloacae*. Antimicrob Agents Chemother 40:2080–2086. doi:10.1128/AAC.40.9.2080. 8878585 PMC163477

[22] Hopkins KL, Findlay J, Doumith M, Mather B, Meunier D, D’Arcy S, Pike R, Mustafa N, Howe R, Wootton M, Woodford N 2017 IMI-2 carbapenemase in a clinical Klebsiella variicola isolated in the UK. J Antimicrob Chemother 72:2129–2131. doi:10.1093/jac/dkx103. 28379381

[23] Naas T, Vandel L, Sougakoff W, Livermore DM, Nordmann P 1994 Cloning and sequence analysis of the gene for a carbapenem-hydrolyzing class A beta-lactamase, Sme-1, from Serratia marcescens S6. Antimicrob Agents Chemother 38:1262–1270. doi:10.1128/AAC.38.6.1262. 8092824 PMC188196

[24] Ambler RP, Coulson AF, Frere JM, Ghuysen JM, Joris B, Forsman M, Levesque RC, Tiraby G, Waley SG 1991 A standard numbering scheme for the class A beta-lactamases. Biochem J 276:269–270. doi:10.1042/bj2760269. 2039479 PMC1151176

