# Supplementary material for: Novel Carbapenemases FLC-1 and IMI-2 Encoded by an Enterobacter cloacae Complex Isolated from Food Products
Source: Antimicrob Agents Chemother. 2019 May 23;63(6):e02338-18. doi: 10.1128/AAC.02338-18 (PMC6535546; doi:10.1128/AAC.02338-18)
Supplement: Supplemental file 1 [file AAC.02338-18-s0001.pdf]

Supplemental Material

Novel carbapenemase FLC-1 and IMI-2 encoded by an *Enterobacter cloacae* complex isolated from food products.

Michael S.M. Brouwer, Kamaleddin H. M. E. Tehrani, Michel Rapallini, Yvon Geurts, Arie Kant, Frank Harders, Vida Mashayekhi, Nathaniel I. Martin, Alex Bossers, Dik J. Mevius, Ben Wit, Kees T. Veldman

Preparation of bacterial cytoplasmic fractions.

Cells were grown in YT2x (0.1% glucose, 50 µg/mL kanamycin). Upon reaching OD_{600}, 0.65, arabinose (0.2% final concentration) was added and after 2 h the cells were harvested by centrifugation at 6000 rpm (20 min, 4 °C). Pellets were resuspended in PBS (0.05% Triton X-100, 150 mM NaCl pH 7.4) and cells disrupted by two freeze-thaw cycles and three 30-second sonication cycles. Cell debris was removed by centrifugation at 12000 rpm (20 min, 4 °C). Protein content of the supernatant was determined by Pierce™ BCA protein assay kit following the manufacturer's protocol.

IC_{50} determination

The inhibitory activity of clavulanic acid against FLC-1 fraction was assessed using nitrocefin as substrate. On a polystyrene 96-well plate and using the assay buffer described above, FLC-1 fraction (2 µg/mL) was incubated with clavulanic acid ranging from 1000 to 0.004 µM for 15 min at 25 °C. Nitrocefin with the concentration corresponding to K_m value (44 µM) was added to all the wells and absorption at 492 nm was monitored over 30 scan cycles. The initial velocity data was normalized using nitrocefin with enzyme in the absence of inhibitor as 100% activity and nitrocefin in the absence of enzyme as 0% activity. IC_{50} curve-fitting was performed on Log(concentration) vs. Activity (%) data using GraphPad Prism 7 software.

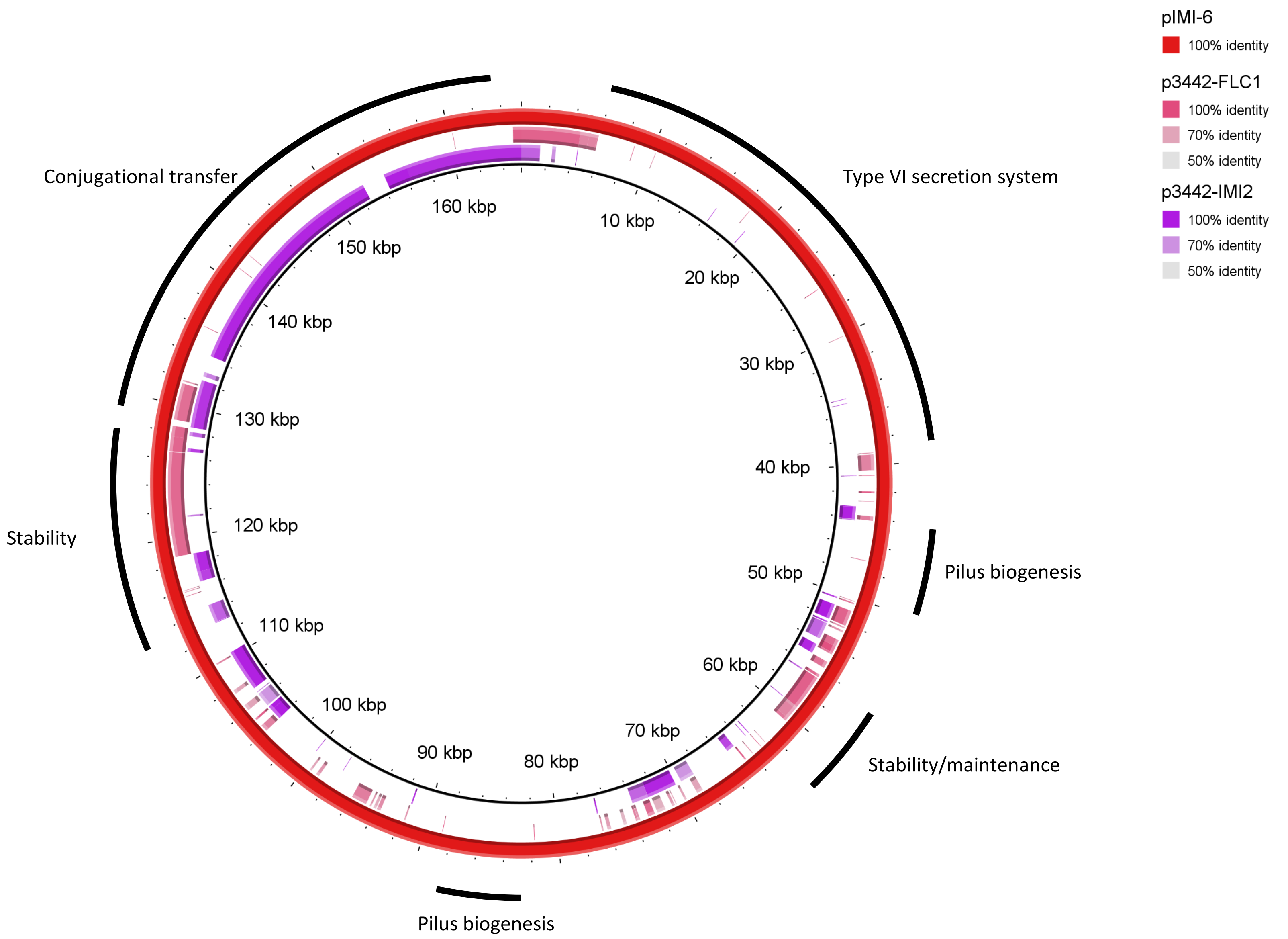

Figure S1 Schematic comparison of plasmids p3442-FRI1 and p3442-IMI2 with pIMI-6 (accession number KX786187). Percentage sequence identity between the plasmids is shown by color gradients. Functional regions of the plasmids are indicated according to Boyd et al. (1).

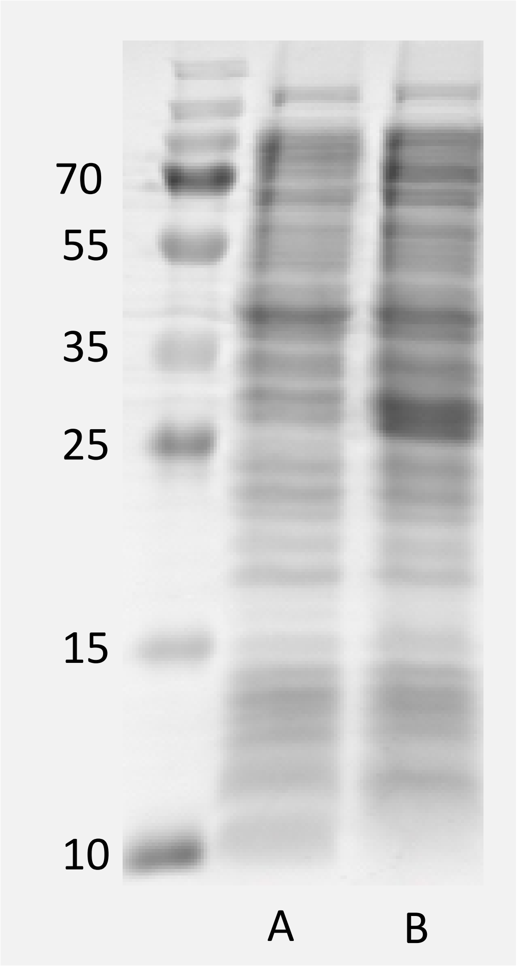

Figure S2 SDS-PAGE gel of *E. coli* LMG-194 pBAD-FLC. Periplasmic protein fractions were loaded before induction (lane A) and after 2 hr induction with 0.2% arabinose (lane B).

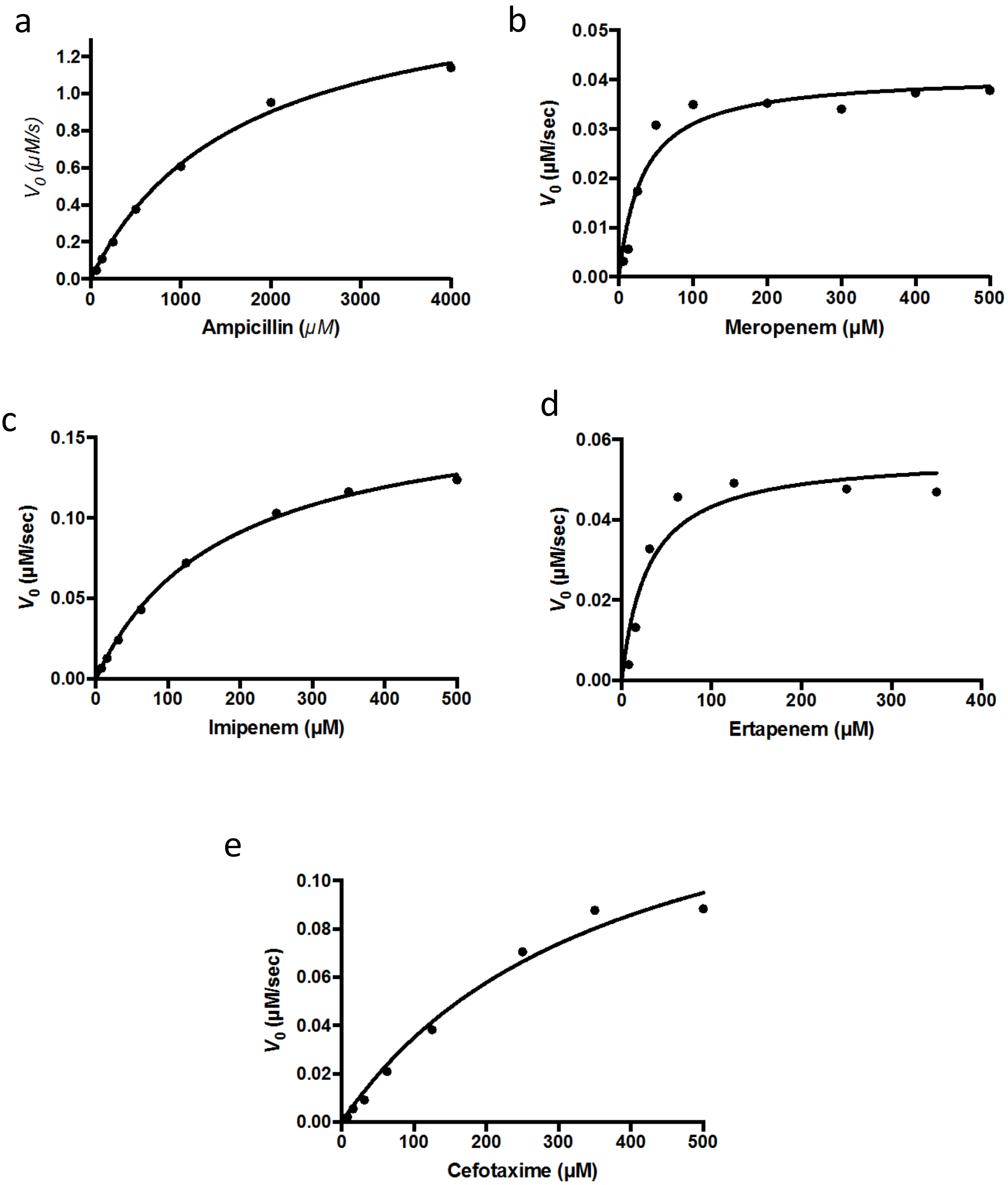

Figure S3 Michaelis-Menten plots of FLC-mediated hydrolysis of selected β-lactam antibiotics: (a) Ampicillin, (b) Meropenem, (c) Imipenem, (d) Ertapenem, (e) Cefotaxime.

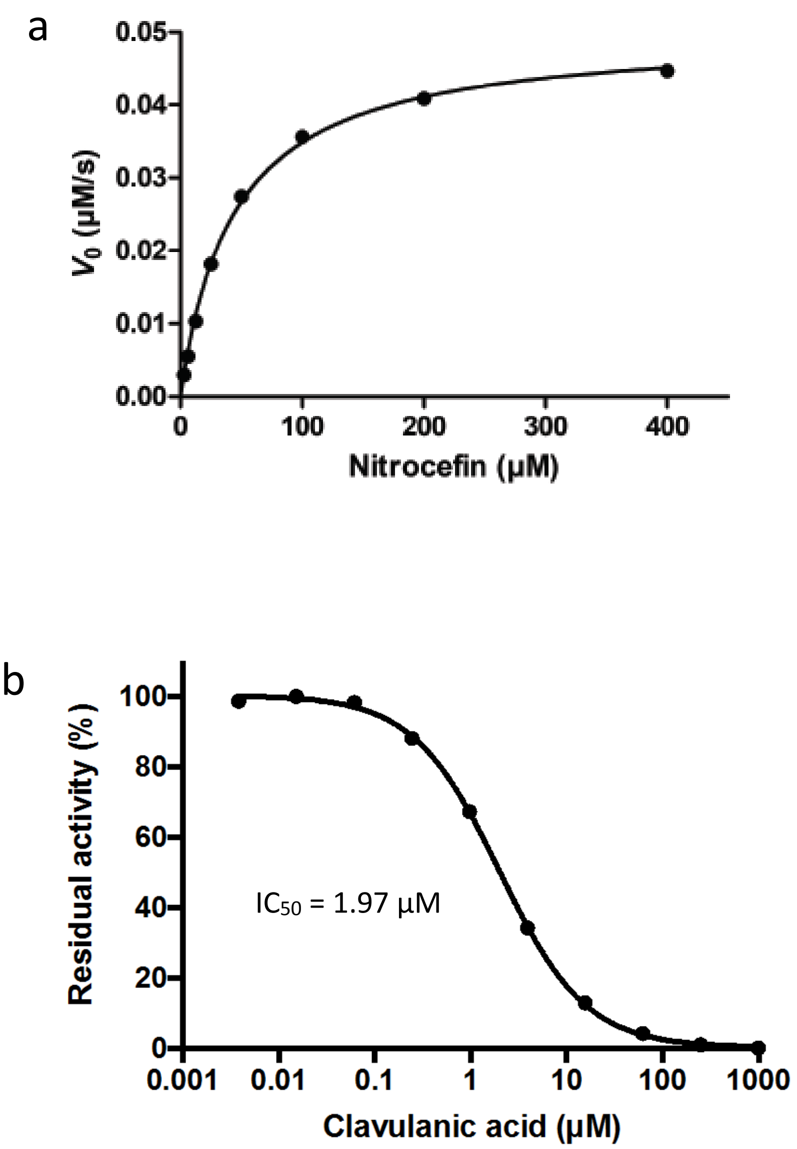

Figure S4 Inhibitory effect of clavulanic acid on FLC-1 enzymatic activity. (a) Michaelis-Menten plot of nitrocefin using 2 μg/mL of FLC-1 protein fraction. V_{max} = 0.055005 μM/s (± 0.0007502), K_m = 43.65 μM (± 2.114). (b) Representative dose-response curve of FLC-1 inhibited by clavulanic acid. IC_{50} = 1.974 μM (± 0.090). Nitrocefin was used as chromogenic substrate.

References to supplementary file.

1 Boyd DA, Mataseje LF, Davidson R, Delport JA, Fuller J, Hoang L, Lefebvre B, Levett PN, Roscoe DL, Willey BM, Mulvey MR. 2017. Enterobacter cloacae Complex Isolates Harboring blaNMC-A or blaIMI-Type Class A Carbapenemase Genes on Novel Chromosomal Integrative Elements and Plasmids. Antimicrob Agents Chemother 61.
